# Neural compensation in adulthood following very preterm birth demonstrated during a visual paired associates learning task

**DOI:** 10.1016/j.nicl.2014.08.009

**Published:** 2014-08-19

**Authors:** Philip J. Brittain, Sean Froudist Walsh, Kie-Woo Nam, Vincent Giampietro, Vyacheslav Karolis, Robin M. Murray, Sagnik Bhattacharyya, Anastasia Kalpakidou, Chiara Nosarti

**Affiliations:** a Department of Psychosis Studies, Neurodevelopment and Mental Health Group, Institute of Psychiatry, King’s College London, De Crespigny Park, London, SE5 8AF, UK; b Department of Neuroimaging, Centre for Neuroimaging Sciences, Institute of Psychiatry, King’s College London, De Crespigny Park, London SE5 8AF, UK

**Keywords:** Very preterm birth, Learning disability, Neuroplasticity, fMRI, Visual paired associates

## Abstract

Very preterm birth (VPT; < 33 weeks of gestation) is associated with an increased risk of learning disability, which contributes to more VPT-born children repeating grades and underachieving in school. Learning problems associated with VPT birth may be caused by pathophysiological alterations in neurodevelopment resulting from perinatal brain insult; however, adaptive neuroplastic processes may subsequently occur in the developing preterm brain which ameliorate, to an extent, the potential sequelae of altered neurophysiology. Here, we used functional magnetic resonance imaging (fMRI) to compare neuronal activation in 24 VPT individuals and 22 controls (CT) in young adulthood during a learning task consisting of the encoding and subsequent recognition of repeated visual paired associates. Structural MRI data were also collected and analysed in order to explore possible structure-function associations. Whilst the two groups did not differ in their learning ability, as demonstrated by their capacity to recognize previously-seen and previously–unseen visual pairs, between-group differences in linear patterns of Blood Oxygenation Level Dependant (BOLD) activity were observed across the four repeated blocks of the task for both the encoding and recognition conditions, suggesting that the way learning takes place differs between the two groups. During encoding, significant between-group differences in patterns of BOLD activity were seen in clusters centred on the cerebellum, the anterior cingulate gyrus, the midbrain/substantia nigra, medial temporal (including parahippocampal) gyrus and inferior and superior frontal gyri. During the recognition condition, significant between-group differences in patterns of BOLD activity were seen in clusters centred on the claustrum and the posterior cerebellum. Structural analysis revealed smaller grey matter volume in right middle temporal gyrus in VPT individuals compared to controls, however volume in this region was not significantly associated with functional activation. These results demonstrate that although cognitive task performance between VPT individuals and controls may be comparable on certain measures, differences in BOLD signal may also be evident, some of which could represent compensatory neural processes following VPT-related brain insult.

## Highlights

• We investigated visual memory ability in young adults who were born very prematurely and a term-born control group
• Similar performance levels were observed between the groups on a task measuring visual memory capacity
• fMRI analysis revealed that there are significantly different processes occurring between the groups concerning the adaptation of neural resources during learning and recall
• Structural differences were also found, with very-prematurely born adults having smaller grey matter volume in a region relevant to visual processing
• In the preterm group, some of these different neural responses may represent the results of compensatory neuroplastic processes which aid in overcoming perinatal brain insult

## 1 Introduction

Very preterm birth (VPT; < 33 completed weeks of gestation) is associated with an increased risk of cognitive disability in childhood and adolescence. Studies have found modest but statistically significant deficits in areas including perceptual–motor skills (Taylor et al., 2000), language ability (Taylor et al., 2000; Rushe et al., 2004; Nosarti et al., 2008), executive functions (Nosarti et al., 2008; Giménez et al., 2006) verbal and visual memory (Taylor et al., 2000; Omizzolo et al., 2014; Caldú et al., 2006) and IQ (Isaacs et al., 2000; Hack et al., 2002). The cognitive deficits resulting from VPT birth are associated with later academic difficulties (Aarnoudse-Moens et al., 2009; Schneider et al., 2004) and potentially have a role in the higher incidence of behavioural and psychiatric difficulties seen in these populations in childhood and adulthood (Johnson, and Marlow, 2011; Nosarti et al., 2012).

The extensive structural brain alterations seen in VPT populations, from infancy through to young adulthood, likely represent an underlying cause of cognitive impairment (Nosarti et al., 2008; Omizzolo et al., 2014; Woodward et al., 2006; Ball et al., 2012). However, recent research on the functional sequelae of VPT birth has led to the hypothesis that adaptive neuroplastic processes may allow some VPT born individuals to attain levels of cognitive functioning which are less deficient than might otherwise have been expected given these pathophysiological occurrences (Giménez et al., 2005; Schafer et al., 2009; Gozzo et al., 2009; Narberhaus et al., 2009; Lawrence et al., 2010; Salvan et al., 2013).

Postulated compensatory neural pathways can be observed when VPT born individuals and controls complete the same behavioural tasks, whilst their brain activity is measured using functional magnetic resonance imaging (fMRI). Studies investigating Blood Oxygen Level Dependent (BOLD) signal fluctuations during tasks involving language functions, for example, have identified altered patterns of neural interconnectivity between task-specific brain areas (i.e. frontal and temporal cortices) in individuals born VPT compared to controls. During a passive auditory language task, stronger connectivity was observed in VPT-born children between left Wernicke’s area and the right inferior frontal gyrus (the homologue of Broca’s area) and the supramarginal gyri bilaterally (Gozzo et al., 2009); whereas during a semantic association task, VPT-born children demonstrated stronger connectivity between typical language processing and sensorimotor areas, whilst also showing weaker connectivity within areas of the prefrontal cortex (Schafer et al., 2009).

Other studies have investigated brain structure and function simultaneously, to elucidate how damage to the former could effect the latter. Salvan et al. (2013) demonstrated functional differences between VPT born young adults and controls using a verbal paired associates learning task. During the task, pairs of word-stimuli were presented four times (encoding), intercalated with four blocks of cued-recall trials. Equivalent performance on the behavioural measures was seen, but between-group differences in patterns of BOLD activity were apparent in the right anterior cingulate-caudate body during encoding and in the thalamus and hippocampus/parahippocampal gyrus during recall. This study also reported reduced white matter integrity in the VPT group in tracts passing through the thalamic/hippocampal region that was differently activated during recall, suggesting that functional activation is partly accounted for by anatomical differences in regions displaying BOLD signal change. In another study, Gimenez and colleagues (Giménez et al., 2005), using a declarative ‘face-name’ memory task, demonstrated significantly greater right hippocampal activation in VPT adolescents than controls, which was associated with a significant decreased volume of the left hippocampus. The authors interpreted this as evidence of a compensatory mechanism in the VPT individuals. Interestingly, in this study, despite the proposed neural compensation, behavioural performance in the VPT group was still poorer than the control group, indicating that such plasticity may not always be fully effective.

Of relevance to this study is a report by Narberhaus et al. (Narberhaus et al., 2009), which used the same visuo-perceptual learning task we investigated and described the mean BOLD signal response during four encoding and four recognition blocks of visual stimuli-pairs in VPT-born adults compared to controls. They reported that, despite no significant performance differences between the groups, during encoding VPT subjects showed increased activation compared to controls in the left caudate nucleus, right cuneus and left superior parietal lobule and a mean decreased signal in the right inferior frontal gyrus. During recognition, VPT individuals showed a mean increased BOLD signal response compared to controls in the right cerebellum and in the anterior cingulate gyrus bilaterally. The authors argued that these differential activation patterns represented neural compensation following perinatal brain injury subsequent to very preterm birth. However, as noted by the authors, an important methodological limitation with their analysis concerned the averaging of activations across the four repeated blocks of the task. This had the potential to mask more ‘pure’ memory effects with repetition effects and retrieval with encoding effects.

Therefore, in this study, we extended the work of Narberhaus et al. (Narberhaus et al., 2009) by reanalysing their data to study the adaptation of neural resources during the learning and recall processes. We aimed to investigate whether the way learning of visual paired associates takes place differs between very preterm-born adults and control participants. In order to explore this we determined brain regions, in a sample of VPT young adults and separately in a control group, that demonstrated either increasing or decreasing linear activation patterns over the four repeated blocks of the encoding and recognition phases. We then looked for any patterns of functional adaptation that differed significantly between the groups.

In normative samples, paired associates learning tasks typically activate a fronto-parieto-occipital network (Neuner et al., 2007), as well as a hippocampal-diencephalic circuitry and medial temporal lobe structures (Neuner et al., 2007; Stark, and Squire, 2000; Strange et al., 2002).

Specifically, decreased activation in thalamus and superior frontal gyrus has been associated with repeated stimuli presentations in humans (Neuner et al., 2007). A possible explanation could be that activation during the first learning blocks reflect a direct access to the associations learnt during encoding which exploit areas of the thalamus projecting to the prefrontal cortex (Klein et al., 2010) and hippocampus (Aggleton et al., 2010). With practice and successful retrieval of the paired associates, subsequent retrieval may become more automatic and thus may not require access to regions centrally involved in encoding.

We hypothesized that VPT born young adults would display altered linear patterns of BOLD signal response compared to controls during the processes of encoding and recognition in components of the fronto-parieto-occipital, hippocampal-thalamic and temporal lobe networks, which include areas found to be particularly impacted in VPT cohorts, both at a functional (Giménez et al., 2005; Narberhaus et al., 2009; Salvan et al., 2013; Scheinost et al., 2014) and a structural level (Nosarti et al., 2008; Ball et al., 2013; Northam et al., 2012; Eikenes et al., 2011). We further conducted exploratory analyses to investigate whether structural alterations (using a whole-brain analysis approach) in the VPT group would be significantly associated with functional activation (Narberhaus et al., 2009; Salvan et al., 2013).

## 2 Materials and methods

### 2.1 Participants

In 1983–84, 147 infants born at less than 33 weeks gestation and admitted consecutively to the Neonatal Unit at University College London Hospital (UCLH) within 5 days of birth, survived, were discharged, and were enrolled for long-term follow up. Of this cohort, 78 individuals were born at 28 or less weeks of gestation and 69 were born between 29 and 33 weeks of gestation. At age 15 years, 113 (76.9%) of these individuals received a cognitive, behavioural and neurological assessment and 90 (61.2%) had an MRI. At age 20, 94 (83.2%) of the individuals assessed in adolescence agreed to participate in follow-up (Allin et al., 2008). For the current fMRI study, 24 young adults of both sexes who were born VPT with no history of cerebral palsy, grade 3/4 intraventricular haemorrhage or periventricular leukomalacia as assessed by neonatal cranial ultrasound, were randomly selected from the cohort described above. These are the same subjects who participated in the study by Narberhaus et al. (2009), with the addition of three VPT individuals who were previously not analysed due to image corruption issues which were successfully fixed prior to this analysis).Twenty-two controls (CT) were recruited from advertisements in the local press and university and were selected according to age, handedness and gender. Inclusion criteria were full-term birth (37–42 completed weeks of gestation); exclusion criteria were birth complications (e.g. low birth weight defined as < 2500 g, endotracheal mechanical ventilation), prolonged gestation (greater than 42 weeks), history of psychiatric illness, severe hearing deficits and motor impairment. All participants were English native speakers and right handed. The experiments were undertaken with the understanding and written consent of each subject, with the approval of the appropriate local ethics committee, and in compliance with national legislation and the Code of Ethical Principles for Medical Research Involving Human Subjects of the World Medical Association (Declaration of Helsinki).

### 2.2 Functional MRI task: Visual paired associates

**Fig. 1:**
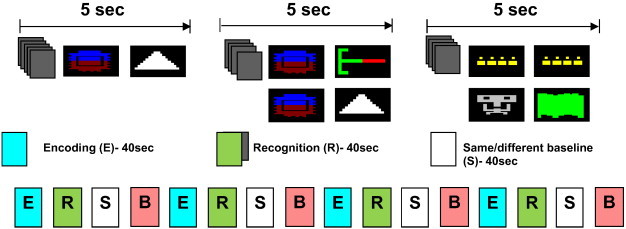
Schematic diagram of the visual stimuli and running order used in the task. The pink ‘B’ represents the low-level baseline condition.

The task used here is identical to that used by Narberhaus et al. (2009) and contained the following conditions: encoding, recognition, same/different discrimination and low-level baseline, presented in that order (see Fig. 1). During the encoding condition, participants were presented with pairs of coloured abstract pictures on black squares and were required to indicate via a button response (‘Yes’/‘No’) if the two pictures were associated. Subjects were posed this question in order that they might think about ways in which the two images were linked, which would require close comparison and thus support encoding of the pairs. The same visual pairs were presented in the encoding condition four times but within each block the order of the picture pairs was randomized. In the recognition condition, one picture from each of the pairs presented during encoding was presented either paired with the same (50% of trials) or with a novel picture (50% of trials). Participants were required to indicate if they had seen those pairs of pictures before (‘Yes’/‘No’). Participants were instructed not to press any button if they did not know what to answer. To reduce the possible confounding effects of differential task performance between the groups on blood oxygen level–dependent signal, in each recognition block of 8 responses each, only activation related to correct responses was modelled.

In the same/different baseline condition, participants viewed pairs of novel pictures which were clearly identical or dissimilar and were required to indicate whether the pictures were the same (‘Yes’/‘No’). There was also a low-level baseline condition during which participants were shown two black rectangles, with no pictures inside, of the same dimension as those containing pictures in the preceding conditions and were instructed to press one of the buttons at random. This condition was subtracted from all subsequent analyses.

Participants were familiarized with the task during an offline training session with two repetitions of the four conditions, each containing 4 stimulus pairs using different pictures to those subsequently presented during the experimental “on-line” session. All stimuli were shown in central vision. Responses during each condition were recorded online. There was no rest (fixation) period between the blocks, but an ‘instruction’ question preceded each encoding (“Are these pictures associated?”), recognition (“Have you seen this pair of pictures before?”), same/different baseline (“Do these pictures look the same?”) and low-level baseline block (“Look at the squares”). Each condition was presented in blocks lasting 40 s, with 8 presentations of stimulus pairs per block. There were 4 repetitions of each block in the same order (encoding, recognition, same/different baseline, low-level baseline).

### 2.3 MRI data acquisition

Image acquisition was performed using a 1.5 T GE Sigma Neurovascular MR system (GE Medical Systems, Milwaukee, WI, USA). A quadrature head coil was used for RF transmission and reception. An inversion recovery EPI data set with 3 mm thick near-axial slices (inter-slice gap .3 mm) and an in-plane resolution of 1.5 mm (TR 3000 ms, TE 40 ms, flip angle 90°) was acquired to facilitate mapping of the functional data into Talairach space. One hundred and forty eight T2*-weighted images were acquired at each of 16 near-axial 7 mm thick planes (inter-slice gap .7 mm) parallel to the intercommissural (AC–PC) plane so as to include the whole brain (FOV 24 × 24 cm and matrix 64^2^), the first four (dummy) volumes being discarded to allow for T1 equilibration effects. The interstimulus interval (ISI) was 5 s. This length was chosen to allow for the longer reaction time latencies of very preterm-born individuals and to provide them with a slightly longer than usual interval of rest between trials (Rose, and Feldman, 1996). The period between the acquisition of clustered image volumes was set at 3.5 s and the image volume acquisition at 1.5 s (TE 40 ms, ﬂip angle 90°). Three-dimensional T_1_-weighted gradient-echo sequences were also collected in order to allow reconstruction in any plane of 124 1.5 mm slices (TR 35 ms, TE 5 ms, flip angle 35°) to enable the measurement of structural MRI data.

### 2.4 Functional MRI analysis

#### 2.4.1 Individual and group mapping

The analysis was hypothesis-driven, and modelled patterns of increase or decrease of activation across the four blocks during encoding, and separately, during recognition. The fMRI data were analysed with the XBAM software (version 4) developed at the Institute of Psychiatry (http://brainmap.co.uk) using a non-parametric permutation-based strategy to minimize assumptions. The non-parametric approach is important to achieve rigorous statistical inference given the difficulty of establishing normality in fMRI data (Hayasaka, 2003; Thirion et al., 2007). The images were first corrected for subject motion (Bullmore et al., 1999a) and then smoothed using a Gaussian filter (FWHM 8.8 mm) chosen to improve signal-to-noise ratio over the spatial neighbourhood of each voxel.

Responses to the experimental paradigm were then detected by fitting a linear model in which each component of the design was convolved separately with two gamma variety functions (peak responses at 4 and 8 s) to allow for variability in the haemodynamic delay. A goodness of fit statistic was computed, consisting of the ratio of the sum of squares of deviations from the mean image intensity due to the model (over the whole time series) to the sum of squares of deviations due to the residuals (SSQ). This addresses the problem inherent in the use of the F statistic that the residual degrees of freedom are often unknown in fMRI time series due to the presence of coloured noise in the signal. The data were permuted by the wavelet-based method described and extensively characterized in Bullmore et al. (Bullmore et al., 2001), which permits the data-driven calculation of the null distribution of SSQ under the assumption of no experimentally-determined response. This distribution can then be used to threshold the activation maps at any desired type I error rate. In addition to the SSQ, the percentage BOLD change was also calculated from the model fit at each voxel. In order to increase sensitivity and reduce the multiple comparison problem, the analysis was extended from voxel to cluster level using a method described in detail in Bullmore et al. (1999b) which has been shown to give excellent cluster-wise type I error control. The observed and randomized SSQ data for each individual were normalized into the standard space (Talairach, and Tournoux, 1988) and group maps of activated regions were computed using the group median as described in Brammer et al. (Brammer et al., 1997). Permutation strategies and median statistics were employed to allow exact computation of *p*-values with minimal assumptions and the minimization of outlier effects. The final cluster maps were thresholded in such a way as to obtain < 0.9 false positive 3D clusters per map.

#### 2.4.2 Group differences

The between-group analysis aimed to find significant interactions between group and linear trend of activation. Comparisons of responses between groups were performed by fitting the data at each intracerebral voxel at which all subjects have non-zero data by linear modelling of the contrast of interest. The model is fitted by minimizing the sum of absolute deviations rather than the sums of squares to reduce outlier effects. The null distribution is computed by permuting data between groups (assuming the null hypothesis of no effect of group membership) and refitting the model at each voxel. Group difference maps at any desired voxel or cluster-wise type I error rate can then be computed by appropriate thresholding of this null distribution. The statistical thresholds were adjusted in such a way as to obtain < 0.9 false positive activated 3D clusters per map.

### 2.5 Structural MRI data processing and analysis

Structural MRI data sets were analysed using voxel-based morphometry (VBM) in Statistical Parametric Mapping SPM8 (Wellcome Department of Cognitive Neurology, Institute of Neurology, London, UK, http://www.fil.ion.ucl.ac.uspm/software/spm8/), running on Matlab 7.8 (Math-Works, Natick, USA). The T1-weighted images were affined-registered to an SPM T1 template and segmented into grey and white matter. Then, the affined-registered grey matter images were normalized to a Montreal Neurological Institute (MNI) coordinate space using the unified DARTEL algorithm (Ashburner, 2007) with modulation for non-linear components, which compares the absolute amount of tissue corrected for individual brain sizes. All grey matter images were smoothed with a 12 mm Gaussian kernel and used for statistical analyses. A whole brain statistical comparison evaluating regional grey matter differences between very preterm born individuals and controls was performed at the statistical threshold of *p* < .05, after family-wise-error (FWE) correction. Grey matter eigenvalues for each study participant were calculated for any clusters where significant between-group differences were noted; SPM’s ‘volume of interest’ (VOI) data extraction tool was employed. The coordinates locating clusters which significantly differed between the two groups were originally reported in MNI space. The ones we are reporting here were converted to Talairach space using the Non-linear Yale MNI to Talairach Conversion Algorithm (http://www.bioimagesuite.org) (Lacadie et al., 2008).

#### 2.5.1 Structure-function associations

In order to explore structure-function associations, we looked for significant correlations (Bonferroni corrected, *p* < 0.005) between the structural eigenvalues extracted from areas that significantly differed between the groups and functional SSQ ratios extracted from the regions demonstrating significant linear BOLD response interaction effects. To characterize the change seen across the four blocks of each encoding and recognition interaction, we created a measure of ‘mean slope change’ for the VPT and CT groups separately using the following formula:

((Block 4 – Block 3) + (Block 3 – Block 2) + (Block 2 – Block 1))/3 

### 2.6 Analysis of Neonatal, socio-demographic and behavioural data

These data were explored with independent samples *t*-tests or their appropriate non-parametric equivalent. Online task performance was assessed with a mixed-design ANOVA.

## 3 Results

### 3.1 Neonatal, socio-demographic and behavioural data

Table 1 displays neonatal, socio-demographic, and behavioural data for study participants. The VPT group and controls did not differ statistically in terms of gender distribution (χ^2^ = 2.1_(43)_, *p* > 0.05), age at assessment (*U* = 238.00, N^1^ = 22, N^2^ = 24, *p* > 0.05, two-tailed), nor in ratings of parental social economic class (χ^2^(42) = 5.57, *p* > 0.05). No significant between-group differences were observed in full-scale IQ (*U* = 137.00, N^1^ = 11, N^2^ = 24, *p* > 0.05, two-tailed), verbal IQ (*U* = 134.00, N^1^ = 11, N^2^ = 24, *p* > 0.05, two-tailed) or performance IQ (*U* = 146.00, N^1^ = 11, N^2^ = 24, *p* > 0.05, two-tailed) as assessed by the Wechsler Abbreviated Scale of Intelligence (WASI) (Wechsler, 1999).

In terms of correct response rates for the online behavioural task, a mixed-design ANOVA demonstrated no main effect of group (*F*(44) = 0.10, *p* > 0.05) but a significant main effect of block (*F*(44) = 4.76, *p* = 0.003) with no significant group × block interaction (*F*(44) = 0.74, *p* > 0.05). Post-hoc analyses revealed that the mean correct response rates to block 2 (5.76, S.D. = 1.57) were higher than to block 1 (4.93, S.D. = 1.40; *t* = 3.37, df = 45, *p* = 0.002) and to block 4 (5.72, S.D. = 1.75) higher than to block 1 (*t* = 3.40, df = 45, *p* = 0.001). These results demonstrate a learning effect, equal across groups, from having the stimulus pairs repeated 4 times.

### 3.2 Functional MRI results

#### 3.2.1 Within group fMRI results

For the controls, during encoding, a linear increase in neural activation over the four blocks was seen in a cluster with peak activation in the right brainstem with decreasing activation seen in the left posterior cerebellum and right middle temporal gyrus. During recognition, increasing activation was seen in the left precuneus and left anterior cerebellum with decreasing activation in the left fusiform gyrus (see Table 2).

For the VPT-born individuals, during encoding, a linear increase in neural activation over the four blocks was seen in a cluster with peak activation in the left anterior cerebellum with decreasing activation seen in the right thalamus. During recognition, increasing activation was seen in the left anterior cerebellum with decreasing activation in the right posterior cerebellum, left superior frontal gyrus, left precuneus, left posterior cingulate and right anterior cingulate (see Table 3).

#### 3.2.2 Between group fMRI results

**Fig. 2:**
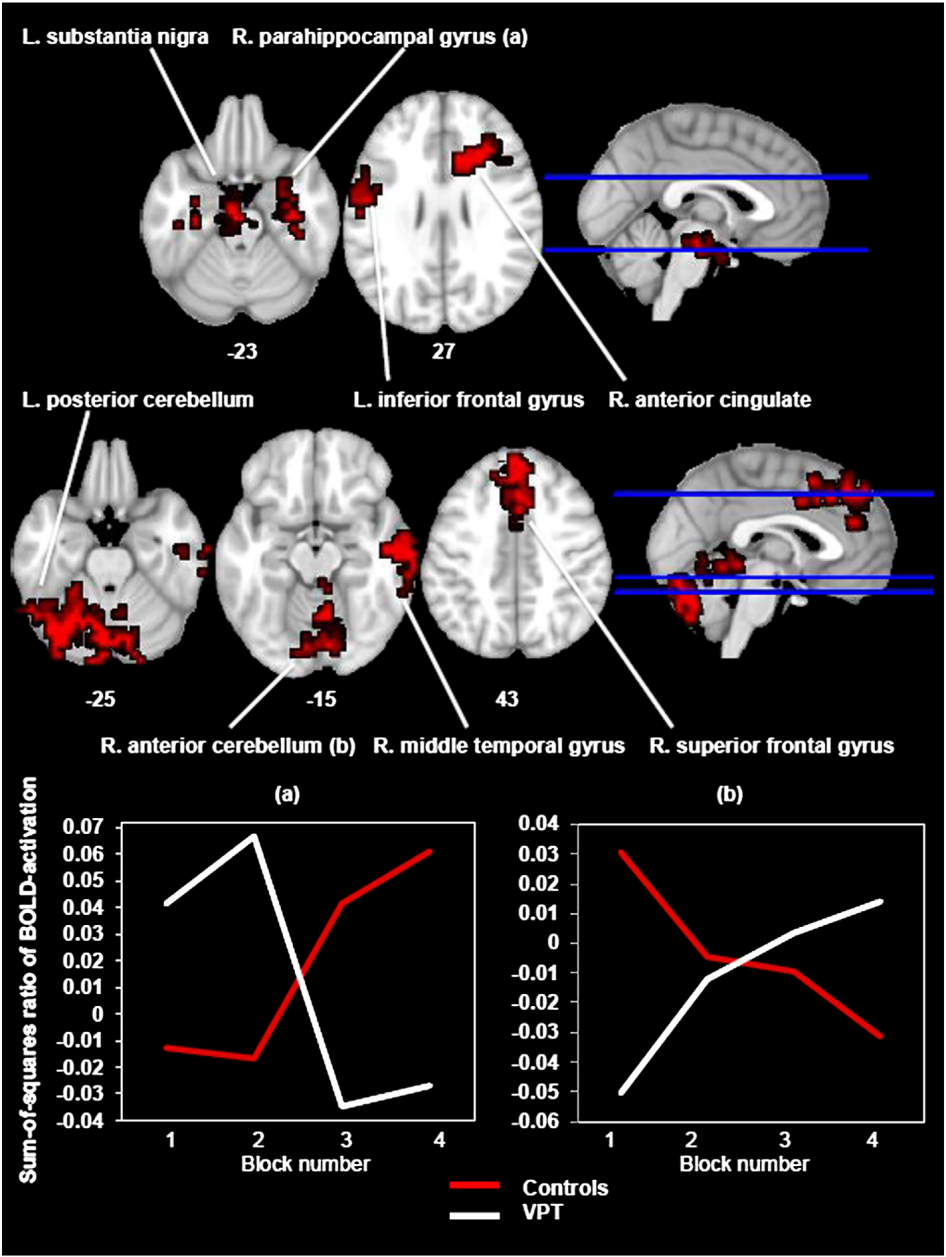
Functional MRI results. Interaction between group and patterns of change of activation during encoding. Axial view showing the four clusters where the CT participants had increasing activation against the decreasing activation of the VPT participants (top row) and the four clusters where the reverse pattern occurred (bottom row). Two representative clusters ((a) right parahippocampal gyrus and (b) right anterior cerebellum) are described further with line graphs.

A statistically significant interaction between group and patterns of change of activation during encoding was found in eight regions. In four of these, the control participants showed an overall increasing pattern of activation in comparison to the VPT participants who showed a decreasing pattern. These clusters had a peak voxel of local maxima in the left midbrain/substantia nigra, the right parahippocampal gyrus, the left inferior frontal gyrus and the right anterior cingulate. A reverse pattern of activation was seen in the remaining four areas, with the VPT participants showing an overall increasing pattern of activation in comparison to a decreasing pattern in the control group. These clusters had a peak voxel of local maxima in the left posterior cerebellum, the right anterior cerebellum, the right middle temporal gyrus and the right superior frontal gyrus (see Fig. 2 and Table 4).

**Fig. 3:**
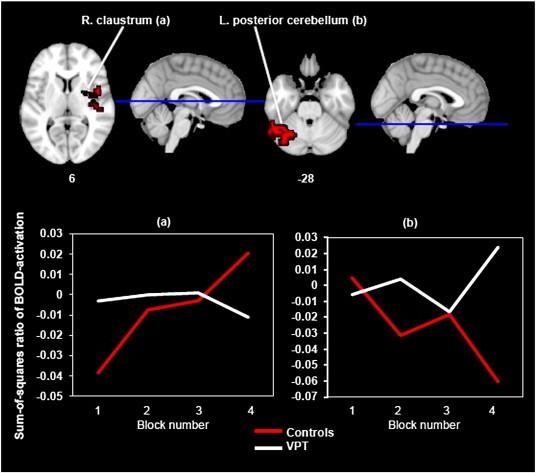
Functional MRI results. Interaction between group and linear change of activation during recognition. Axial view showing the one cluster where the CT participants had increasing activation against the decreasing activation of the VPT participants (left) and the one cluster where the reverse pattern occurred (right). The two clusters ((a) right claustrum and (b) left posterior cerebellum) are described further with line graphs.

A statistically significant interaction between group and patterns of change of activation during recognition was found in just two regions. In one of these, that had a peak voxel of local maxima in the right claustrum, the CT participants showed an overall increasing pattern of activation in comparison to the VPT participants who showed an overall decreasing pattern. A reverse pattern of activation was seen in a cluster with a peak voxel of local maxima in the left posterior cerebellum, with the VPT participants showing an overall increasing pattern of activation in comparison to the CT participants who showed a decreasing pattern (see Fig. 3 and Table 4).

### 3.3 Structural analysis results

Structural analysis revealed smaller grey matter volume in the right middle temporal gyrus (rMTG) in the VPT group compared to CT participants (*x* = 45, *y* = −17, *z* = –30; *p* < 0.001).

#### 3.3.1 Structure-function associations

When eigenvalues extracted from the significant cluster centred in right middle temporal gyrus were correlated with SSQs extracted from the 10 regions where significant linear interactions were found (encoding and recognition), no significant associations were found, after Bonferroni corrections, for the VPT nor CT groups.

## 4 Discussion

Very preterm birth has been associated with long-lasting cognitive deficits, which can have profound effects on academic and career achievement, and are likely to be mediated by structural and functional brain alterations following perinatal brain injury. A recent hypothesis predicts that adaptive neuroplasticity in the developing brain may ameliorate the severity of these deficits in some cases via the utilization of compensatory neural pathways (Giménez et al., 2005; Schafer et al., 2009; Gozzo et al., 2009; Narberhaus et al., 2009; Lawrence et al., 2010; Salvan et al., 2013). One study, by Narberhaus et al. (2009), using an fMRI based visual paired associates task, concluded that the differential patterns of neural activation seen in the VPT group, including decreased BOLD response in inferior frontal gyrus accompanied by increased response in caudate nucleus, cuneus and superior parietal lobule, likely represented compensatory neural processes for the adult consequences of perinatal brain injury. However, a methodological limitation of that study concerned the averaging of activations across the four repeated blocks of the experiment, which masked the underlying neurobiology taking place during learning and recall. By expanding the cohort and reanalysing the data via a linear analysis of the BOLD response over the four repeated blocks of the encoding and recognition phases, we could, for the first time, investigate patterns of functional adaptation in relation to visual learning strategies in VPT-born individuals and controls in young adulthood. The results we found with this improved methodology were substantially different from those of Narberhaus et al. (2009); our analysis highlighted a number of memory and other domain-general cognitive and attentional regions differentially activated between the CT and VPT groups that were not shown in that study. This suggests that a linear analysis approach is more sensitive for detecting functional alterations during a repeated-block learning paradigm than is the method of using mean activation.

The VPT and control groups demonstrated a similar improvement in mean accuracy scores across the four recognition blocks of the online behavioural task. This demonstrated that a learning effect took place. The fMRI results demonstrated that it is the way in which this learning effect takes place that is different between the groups. The first piece of evidence for this is seen in the results from the within group analyses (Tables 2 and 3). This revealed that both groups demonstrated patterns of BOLD signal change across the four blocks of the task in domain-general cognitive and attentional regions typically activated during tasks involving learning (Chein, and Schneider, 2005; Kelly, and Garavan, 2005). However, both groups also showed activation in areas previously demonstrated to be specifically involved in memory processing and, crucially, these were largely different between the groups. For example, during encoding, controls demonstrated increasing activation in the right middle frontal gyrus, which has been associated with visual working memory (Mohr, Goebel, and Linden, 2006; Protzner, and McIntosh, 2007) whereas the VPT group had increasing activation in the left parahippocampal gyrus, which has been associated specifically with non-verbal memory processing (Köhler et al., 1998). It is this differential activation in memory-related regions, seen here and explored further in the between-subjects analysis, which is suggestive of neuroplastic adaptation in the VPT group.

For the between-subjects analysis, we were specifically interested in the interaction between group and block. That is to say, we wanted to investigate whether there were any brain regions that would show significantly different patterns of linear BOLD signal change between groups across the four encoding and, separately, across the four recognition blocks. Eight regions (Table 4) demonstrated such an interaction during the encoding blocks; in four of these there was a pattern of overall progressively increasing activation in the CT participants and decreasing activation in the VPT participants and, in four, the reverse pattern occurred. There were two regions that demonstrated a significant interaction during the recognition blocks; in one of these, activation increased in the CT group and decreased in the VPT group, whilst in the other, the reverse pattern occurred. From these 10 regions, several stand out in relation to our hypothesis of neural adaptation during visual learning in the VPT brain. During the encoding phase, the VPT group had decreasing activation compared to the CT group’s increasing activation in a cluster centred on the right parahippocampal gyrus extending to the hippocampus, key memory-related areas (Stark, and Okado, 2003), as well as in the thalamus which has reciprocal connections to the hippocampus and is central to episodic memory processing (Aggleton et al., 2010) and which together represent components of a fronto-subcortical network involved in associative learning (Lockhart et al., 2012). However, the VPT group showed increasing activation compared to the CT group’s decreasing activation in the right superior frontal gyrus, a region known to be involved in various memory functions including episodic and working memory (Burianova, and Grady, 2007; Cohen et al., 1997; Rypma et al., 1999) which typically decreases in activation with repeated stimuli presentations (Neuner et al., 2007).

In terms of the structural findings, in a whole brain analysis we found the VPT group to have reduced volume, compared to controls, in a cluster that included most of the right middle temporal gyrus. This result is consistent with the findings of a structural study from our group in a larger sample of VPT participants in mid-adolescence, which included the current study subjects (Nosarti et al., 2008), and reported decreased volume in middle temporal gyri bilaterally. However, despite findings in previous studies which observed significant associations between regional volume alterations and BOLD signal change in similar samples of VPT born individuals completing paired associate learning tasks (Narberhaus et al., 2009; Lawrence et al., 2010, Salvan et al., 2013), in the current investigation, after correcting for multiple comparisons, no significant associations between the volume of the right middle temporal gyrus and fMRI results were observed. This lack of association is difficult to account for. However, it may be that despite our best efforts to create a metric which represented the change in BOLD response across the four time points (see section 2.5.1.), inter-subject variation, in relation to precisely when a structural reduction effects BOLD response, could render correlation between a static time-point (i.e. the eigenvalues representing rMTG volume) and a time-series, ineffective.

In relation to our principal hypothesis of neural adaptation during visual learning in the VPT brain, we therefore propose that the increased BOLD response of the VPT subjects, in comparison to the controls, in the right superior frontal gyrus, represents compensatory neural adaptation in response to reduced activity in the right parahippocampal gyrus, hippocampus and thalamus. Furthermore, that the increasing BOLD response in the VPT group, compared to controls, in the right middle temporal gyrus, an area which has been associated with visual semantic processing (Visser et al., 2012), may represent an attempt at compensatory activity in this structurally altered area.

It is likely that much of the differential activation we observed was not related to compensatory neural processes but reflects altered neural responses which, were the subjects to undertake a more challenging task, could potentially result in behavioural differences. Closer inspection of our results supports this. Firstly, the between-subjects analysis revealed three clusters (two during encoding and one during recall) where activation in the cerebellum increased for the VPT subjects but decreased for the controls. Emerging evidence places the cerebellum within a network of regions demonstrating reduced activity with practice during task domain-general activity (Chein, and Schneider, 2005), possibly due to neuronal adaptation to the repeated stimuli (Chein, and Schneider, 2005; Larsson, 2012). The patterns of decreasing cerebellar activation observed in our control group support this theory, whereas the progressively increasing activation in our VPT group suggests that they may have experienced greater difficulty in completing the task.

A second example of altered neural response in the VPT group can be seen in the activation of cingulate cortex (Table 4; encoding region 4, recognition region 1). The anterior cingulate receives input from midbrain dopaminergic neurons (Paus, 2001) which are crucial for motivational salience (Berridge, 1998). Although Narberhaus et al. (Narberhaus et al., 2009) showed that in terms of mean activation across the blocks, VPT individuals activate the anterior cingulate and surrounding areas more than controls, our linear analysis revealed that, over time, the control participants increase their activation in this area, whilst in VPT participants it decreases. This possibly reflects a reduction in motivation as the task progresses. A reduction in task interest, albeit at a neural and not behavioural level, would also explain the decreasing activation of the substantia nigra in the VPT participants, as this region is also important in maintaining stimulus engagement (Reynolds, Hyland, and Wickens, 2001). As stressed previously however, if the examples cited above (cerebellum, anterior cingulate, substantia nigra) do reflect altered, or perhaps ‘sub-optimal’ neural responses in the VPT group, these would likely only become relevant in a task with higher cognitive demands.

There are two other points from our analysis which merit further consideration. Firstly, most of the between-group differences occurred during the encoding and not the recognition phases of the task. This could suggest that the learning of visual-paired associations represents a more neuroanatomically vulnerable process to VPT-related brain insult than recognition. Secondly, if these results do indeed reflect compensatory neurodevelopment, this has occurred even in the absence of serious perinatal brain injury, for which this cohort of VPT individuals screened negative.

There are several limitations to the current study. Firstly, we used a relatively small cohort, which restricts the generalizability of our conclusions. Secondly, the lack of more comprehensive offline visuo-spatial memory assessments in this sample leaves us with the probability that more challenging tasks would highlight behavioural deficits in the preterm cohort which would alter the interpretation of the putative adaptive differences from being fully- to only partially-effective. Thirdly, whilst other research has reported differing patterns of neuronal activation between VPT groups and full-term controls whilst performing paired-associates learning tasks, and used the results to support the hypothesis of compensatory neural adaptation (Giménez et al., 2005; Narberhaus et al., 2009; Salvan et al., 2013; Kalpakidou et al., 2012), it is difficult to draw meaningful comparisons between our findings and those of the other studies, due to the differing nature of the behavioural tasks and analysis methods used. Specifically, our study is the first to use a linear analysis approach to investigate functional differences occurring during a visual paired associate task.

## 5 Conclusions

In conclusion, the above results demonstrate that equivalent behavioural performance between VPT subjects and full-term controls can be underlined by significantly different neural processes. An emerging theory in preterm research is that brain insult in the perinatal period can lead to compensatory neurodevelopmental processes resulting in cognitive functioning being less deficient than might otherwise have been expected (Giménez et al., 2005; Schafer et al., 2009; Gozzo et al., 2009; Narberhaus et al., 2009; Lawrence et al., 2010; Salvan et al., 2013). Whilst the results from this analysis support that theory, other altered neural processes are still evident which could have consequences where behavioural performance requirements are more demanding.

## Figures and Tables

**Table 1:** Neonatal, socio-demographic and behavioural data.

	Preterm (*n* = 24)	Controls (*n* = 22)
Gestational age in weeks (mean, SD)	28.58 (2.08)	−
Birth weight in grams (mean, SD)	1286.88 (391)	−
Gender (female/male)	9/15	13/9
Age in years at assessment (mean, SD)	20.09 (0.58)	20.1 (1.68)
Socio-economic status^a^ (percent)		
I−II	37.5%	31.8%
III	54.2%	31.8%
IV−V	8.3%	36.4%
WASI full-scale IQ (mean, SD)	100.50 (11.80)	103.09 (14.75)
WASI verbal IQ (mean, SD)	96.79 (12.09)	99.45 (13.65)
WASI performance IQ (mean, SD)	103.92 (12.04)	106.36 (15.86)
Correct responses [max. 8] (Mean, SD)		
Block 1	4.92 (1.25)	4.95 (1.59)
Block 2	5.75 (1.39)	5.77 (1.77)
Block 3	5.67 (1.86)	5.09 (1.80)
Block 4	5.71 (1.78)	5.73 (1.75)

a Socio economic status was measured by Her Majesty’s Stationary Office Standard Occupational Classification criteria [Her Majesty’s Stationery Office (HMSO), 1991]. The following SES bands were used: I−II = Managerial and Professional; III = Intermediate (e.g. small employers and own account workers); IV−V = Working (e.g. lower supervisory and technical occupations, semi-routine & routine occupations).

**Table 2:** Group activations for controls during encoding and recognition of visual paired associates. The coordinates of the cluster maxima (the most activated voxel within the cluster) are detailed.

Condition/direction	Cerebral region (Brodmann’s area)	Talairach coordinates			Cluster size	Cluster *p* value
		*X*	*Y*	*Z*		
Encoding/increase	Right brainstem Extends to precentral gyrus, middle frontal gyrus, superior temporal gyrus, posterior cerebellum, inferior frontal gyrus, anterior cerebellum, middle temporal gyrus, left brain stem	7	−26	−40	783	0.0002
Encoding/decrease	Left posterior cerebellum Extends to precuneus, cuneus, middle temporal gyrus, inferior temporal gyrus	−25	−67	−18	1096	0.0002
	Right middle temporal gyrus (20) Extends to inferior parietal lobule, postcentral gyrus, temporal lobe, superior temporal gyrus	47	−33	−7	118	0.0065
Recognition/increase	Left precuneus (31) Extends to paracentral lobule, parietal lobe, precuneus, frontal lobe, cingulate gyrus, inferior parietal lobule	−14	−44	42	155	0.0015
	Left anterior cerebellum Extends to brainstem, temporal lobe, limbic lobe, posterior cerebellum, parahippocampal gyrus, temporal lobe, right brainstem	−25	−30	−24	98	0.0051
Recognition/decrease	Left fusiform gyrus (37) Extends to superior parietal lobule, posterior cerebellum, middle temporal gyrus, superior temporal gyrus, cuneus	−54	−56	−18	787	0.0002

**Table 3:** Group activations for VPT individuals during encoding and recognition of visual paired associates. The coordinates of the cluster maxima (the most activated voxel within the cluster) are detailed.

Condition/direction	Cerebral region (Brodmann’s area)	Talairach coordinates			Cluster size	Cluster *p* value
		*X*	*Y*	*Z*		
Encoding/increase	Left anterior cerebellum Extends to fusiform gyrus, parahippocampal gyrus, brainstem, posterior cerebellum	−14	−37	−24	88	0.0059
Encoding/decrease	Right thalamus Extends to inferior parietal lobe, frontal lobe, posterior cingulate gyrus, temporal lobe, brainstem, postcentral gyrus	25	−26	15	188	0.0031
Recognition/increase	Left anterior cerebellum Extends to brainstem, temporal lobe, limbic lobe, posterior cerebellum, parahippocampal gyrus, temporal lobe, right brainstem	−18	−41	−29	66	0.0053
Recognition/decrease	Right posterior cerebellum Extends to lingual gyrus, fusiform gyrus, left cuneus, left posterior cerebellum	36	−63	−40	185	0.0021
	Left superior frontal gyrus (9) Extends to middle/medial frontal gyri, anterior cingulate gyrus	−11	52	20	156	0.0021
	Left precuneus (7) Extends to superior parietal lobule, cuneus	0	−74	42	137	0.0037
	Left posterior cingulate (30) Extends to cingulate gyrus, thalamus, posterior cingulate, temporal lobe, right posterior cingulate	−18	−48	9	106	0.0045
	Right anterior cingulate (32) Extends to cingulate gyrus, frontal lobe, superior frontal gyrus, middle frontal gyrus	18	26	20	87	0.0047

**Table 4:** Differences in brain activation (as indexed by the median SSQ ratio (sum of squares ratio)) between VPT participants and controls during encoding and recognition of visual paired associates.

Condition	Cerebral region (Brodmann’s area)	Talairach coordinates			Cluster size	Cluster *p* value	Signal direction
		*X*	*Y*	*Z*			
Encoding	1. Left posterior cerebellum Extends to inferior temporal gyrus, middle occipital gyrus, fusiform gyrus, lingual gyri bilaterally, parahippocampal gyrus, right anterior cerebellum, right inferior occipital gyrus	−25	−63	−18	285	0.0001	VPTs increasing/controls decreasing
	2. Right superior frontal gyrus (9) Extends to anterior cingulate gyri bilaterally, medial frontal gyri bilaterally	4	48	31	99	0.0015	VPTs increasing/Controls decreasing
	3. Left substantia nigra Extends to thalamus, brainstem bilaterally, insula, parahippocampal gyrus, superior temporal gyrus	−7	−22	−13	94	0.0012	Controls increasing/VPTs decreasing
	4. Right anterior cingulate (32) Extends to insula, frontal lobe	18	26	20	64	0.0022	Controls increasing/VPTs decreasing
	5. Right anterior cerebellum Extends to cuneus, lingual gyrus, parahippocampal gyri bilaterally, left anterior cerebellum, left posterior cingulate, left cuneus, left lingual gyrus	7	−59	−2	52	0.0037	VPTs increasing/Controls decreasing
	6. Right parahippocampal gyrus (36) Extends to anterior cerebellum, lentiform nucleus, fusiform gyrus, hippocampus	32	−26	−24	50	0.0058	Controls increasing/VPTs decreasing
	7. Right middle temporal gyrus (21) Extends to superior / middle temporal gyri,	61	−26	−13	43	0.0037	VPTs increasing/Controls decreasing
	8. Left inferior frontal gyrus (9) Extends to precentral/postcentral gyri, middle frontal gyrus	−51	4	26	39	0.0061	Controls increasing/VPTs decreasing
Recognition	1 Right claustrum Extends to insula, transverse temporal gyrus, inferior/medial frontal gyrus, inferior parietal lobule, cingulate gyrus	32	0	9	142	0.0003	Controls increasing/VPTs decreasing
	2 Left posterior cerebellum Extends to middle / superior temporal gyri, fusiform gyrus, middle occipital gyrus, lingual gyrus	−25	−59	−18	125	0.0005	VPTs increasing/Controls decreasing
